# Placing a Priority on Downtime

**DOI:** 10.31486/toj.23.5034

**Published:** 2023

**Authors:** Ronald G. Amedee

**Affiliations:** Director of Clinical School and Professor, The University of Queensland Medical School, Ochsner Clinical School, New Orleans, LA; Editor-in-Chief, *Ochsner Journal*

*Everything good, everything magical happens between the months of June and August.*

–*Jenny Han*

The Summer 2023 issue of the *Ochsner Journal* contains five original research articles, one review and contemporary update, and four case reports and clinical observations which serve as the principal elements for this edition. In addition, please consider reading the three editorials in this issue entitled “The Case for Understanding Interdisciplinary Relationships in Health Care,” “Transparency in Hospital Medicine Metrics: An Effective Approach to Hospital Medicine Practice Management and Influencing Positive Behavior for Everyone's Benefit,” and “The Eyeball Test.” This issue also contains the second Health, Medicine, and Society quarterly column, authored by Price-Haywood, an Ochsner clinician-scientist.

This issue's original research includes articles by Smith, Peairs, and Nossaman on “Rate of Intraoperative Crystalloid Administration During Thoracic Surgery Is Causal in Reducing Postoperative Hospital Length of Stay,” followed by “Impact of Frailty Upon Surgical Decision-Making for Left-Sided Colon Cancer” authored by Sibia, Badve, Istl, et al. Next, please find work by Schaible, Langhals, Taylor, et al detailing “Residency Experience With Physical Examination– and Ultrasound-Indicated Cerclage: A Single Center Retrospective Study,” followed by “Change in Methicillin-Resistant *Staphylococcus aureus* Testing in the Intensive Care Unit as an Antimicrobial Stewardship Initiative” by Smith, DuMontier, Bushman, et al. Finishing out the original research articles in this issue is “Sinonasal Complications Following the Sinus Lift Procedure” authored by Fischer, Riley, and Kacker.

The review and contemporary update in this issue is the “Impact of Coffee Consumption on Cardiovascular Health” by Mendoza, Sulague, Posas-Mendoza, and Lavie.

Case reports and clinical observations in this issue include “Cooled Radiofrequency Ablation for Intercostal Neuralgia” by Fiala, Martens, Keith, et al, followed by “Acrometastasis in Breast Carcinoma” by Galliano, Bragg, Rangani, and Froom. Rounding out this section, Lally and Galarneau submit “Psychiatric Manifestations as the Primary Presentation of Frontal Meningioma,” and “Thoracic Spinal Stenosis From Calcified Ligamentum Flavum” is offered by Cavazos, Schultz, Higginbotham, and Vaidya.

As far as good and magical things happening during the summer months, as health care workers we should prioritize downtime to practice well-being and enhance personal resilience. This can come in the form of a week(s)-long vacation, frequent extended weekends, or simply a day off work here and there throughout the coming months. The only way we can continue to take excellent care of our patients is if we take deliberate and meaningful care of ourselves. Our time in academic and/or clinical medicine is not a short race but a lifelong marathon.

